# Chimeric Antigen Receptor T Cell Therapy in Systemic Lupus Erythematosus: Mechanisms, Clinical Advances, and Future Directions a Comprehensive Review

**DOI:** 10.1007/s12016-025-09114-6

**Published:** 2025-11-27

**Authors:** Ahmad Matarneh, Bayan Matarneh, Omar Salameh, Abdelrauof Akkari, Nasrollah Ghahramani, Naman Trivedi

**Affiliations:** 1 Department of Nephrology, Penn State Milton S. Hershey Medical Center, Hershey, PA USA; 2 Detroit Medical Center, Children’s Hospital, Detroit, MI USA; 3 University Health Truman Medical Center, Kansas City, MO USA

**Keywords:** Systemic lupus erythematosus, Chimeric antigen receptor T cells, CAR-T, CD19, BCMA, B cell depletion, Autoimmunity, Immunotherapy

## Abstract

Systemic lupus erythematosus (SLE) is a chronic, multisystem autoimmune disorder characterized by loss of self-tolerance, immune complex deposition, and progressive organ damage. Despite advances in immunosuppressive therapy, a subset of patients develops treatment-resistant or refractory manifestations; terms used variably in the literature to describe inadequate response to multiple standard immunosuppressants. Chimeric antigen receptor T cell (CAR-T) therapy, a revolutionary modality in oncology, is now emerging as a promising approach in severe autoimmune diseases including SLE. By redirecting autologous T cells to target B cell antigens such as CD19 or BCMA, CAR-T therapy enables deep and sustained B cell depletion, potentially resetting immune tolerance.Early case series have reported encouraging remission rates and serologic improvements in refractory SLE; however, these observations derive from small, uncontrolled studies. The long-term durability, relapse risk, safety profile, and cost-effectiveness of CAR-T therapy in autoimmune disease remain uncertain and require confirmation in larger, controlled trials. This narrative review synthesizes the current understanding of CAR-T therapy in SLE, covering immunopathogenesis, rationale for B cell targeting, CAR-T mechanisms, preclinical evidence, clinical outcomes, safety considerations, and future directions. We integrate data from peer-reviewed studies, conference abstracts, and preprints up to August 2025, and propose a framework for integrating CAR-T into the treatment paradigm for refractory SLE.

## Introduction

Systemic lupus erythematosus (SLE) is a heterogeneous autoimmune disease with variable clinical manifestations ranging from mild cutaneous involvement to life-threatening renal, neurological, or hematologic complications [1, 2]. Over the past two decades, improved disease recognition, supportive care, and the introduction of targeted biologics such as belimumab and anifrolumab have modestly improved outcomes [3]. However, a subset of patients—often termed refractory or treatment-resistant—remains unresponsive to conventional immunosuppressants and biologics, experiencing persistent disease activity, cumulative organ damage, and reduced quality of life [4]. There is no universally accepted definition for refractory SLE. Most studies define refractoriness pragmatically as failure to achieve sustained disease control despite at least two standard immunosuppressants or biologics at adequate dose and duration [3, 4]. This heterogeneity complicates comparison across studies and emphasizes the need for standardized response definitions in future clinical trials.

B cell dysregulation is central to SLE pathogenesis, with autoreactive B cells contributing to autoantibody production, antigen presentation, and pro-inflammatory cytokine release [5]. While B cell depletion strategies such as rituximab have demonstrated benefit in some patients, incomplete depletion, resistance, and eventual relapse limit their long-term efficacy [6, 7]. This has fueled interest in novel, more potent approaches to eliminate autoreactive B cells and reprogram immune tolerance.

Chimeric antigen receptor T cell (CAR-T) therapy, which has transformed outcomes in hematologic malignancies, is now being explored in autoimmune diseases [8]. The rationale for CAR-T in SLE lies in its ability to induce profound and durable B cell depletion, often beyond what is achievable with monoclonal antibodies, while promoting immune reconstitution devoid of autoreactivity [9]. Since the first landmark report by Mackensen et al. in 2022 describing five refractory SLE patients achieving sustained remission after anti-CD19 CAR-T therapy [10], several studies and case series have expanded the evidence base [11–14]. These early results suggest that CAR-T therapy may offer a disease-modifying, possibly curative, intervention for severe SLE.

This review synthesizes the current literature on CAR-T therapy in SLE, including mechanistic rationale, preclinical data, clinical trial outcomes, safety considerations, and future directions. We include peer-reviewed publications, conference abstracts, and preprints to capture the most up-to-date evidence as of August 2025.

### Search Strategy and Scope of Review

The literature search was conducted using PubMed, Scopus, and Web of Science databases for publications between **January 2018 and August 2025**, using the terms “CAR T” OR “chimeric antigen receptor T” AND (“lupus” OR “systemic lupus erythematosus” OR “autoimmune disease”). Reference lists of relevant articles and preprints were also screened manually to identify additional studies, including recent congress abstracts (EULAR 2024, ACR 2024). Only English-language articles were included. This review integrates peer-reviewed studies, conference abstracts, and registered clinical trials to provide a comprehensive and current perspective on CAR T-cell therapy in SLE and related autoimmune diseases.

## Immunopathogenesis of SLE and the Rationale for B Cell Targeting

SLE arises from a complex interplay of genetic susceptibility, epigenetic modifications, environmental triggers, and hormonal influences [15]. Central to its pathogenesis is the breakdown of immune tolerance, particularly involving B and T lymphocytes [16]. Autoreactive B cells contribute to disease through several mechanisms:

Autoantibody production — generating pathogenic immunoglobulins such as anti-double-stranded DNA (anti-dsDNA) and anti-Smith antibodies that form immune complexes [17]. Antigen presentation — B cells present autoantigens to CD4⁺ T cells, promoting further immune activation [18]. Cytokine secretion — including IL-6, TNF-α, and lymphotoxin, which sustain inflammation and tissue injury [19].

In addition, the uptake of immune complexes containing nucleic acids by plasmacytoid dendritic cells through Fcγ and Toll-like receptors triggers a robust type I interferon (IFN-α/β) response, which amplifies B- and T-cell activation and sustains chronic autoimmunity. Type I IFN gene signatures correlate with disease activity and may influence responsiveness to B-cell-directed therapy, underscoring the complex crosstalk between innate and adaptive immunity in SLE pathogenesis [20].The pivotal role of B cells is underscored by the efficacy of B cell-directed therapies in SLE. However, limitations of current agents are evident. Rituximab, an anti-CD20 monoclonal antibody, depletes circulating B cells but spares long-lived plasma cells and tissue-resident B cells [21]. Belimumab, an anti-BAFF antibody, reduces B cell survival signals but achieves only partial disease control in many patients [22]. Moreover, both approaches often result in incomplete or transient depletion, with repopulation of autoreactive B cells preceding relapse [23].

By contrast, CAR-T therapy offers the potential for near-complete depletion of CD19-expressing B cells, including those resistant to antibody-mediated clearance [24]. In oncology, this strategy has led to durable remissions in B cell malignancies; in autoimmunity, it may “reset” the B cell compartment, allowing repopulation by non-autoreactive clones [25].

While immune complex deposition and complement activation remain the dominant pathogenic mechanisms in lupus nephritis and systemic inflammation, several autoantibodies exert pathogenicity independently of immune complex formation. Antiphospholipid antibodies promote thrombosis through direct endothelial activation, platelet aggregation, and complement-independent vascular injury. Antiplatelet antibodies and anti–red cell antibodies mediate peripheral cytopenias via opsonization and Fc-mediated clearance, while anti–NMDA receptor antibodies may contribute to neuropsychiatric lupus through direct receptor modulation. These examples underscore the immunologic heterogeneity of SLE and illustrate that disease manifestations arise from both immune complex–mediated and direct effector antibody mechanisms.

## Principles of CAR-T Cell Therapy

**Fig. 1:**
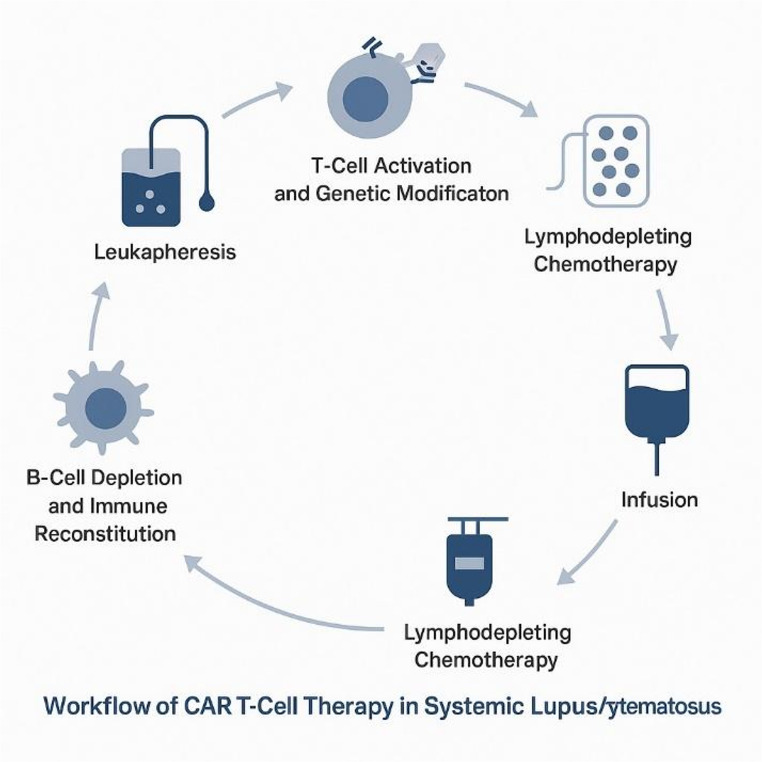
Workflow of CAR T-Cell therapy in systemic lupus erythematosus

**Fig. 2:**
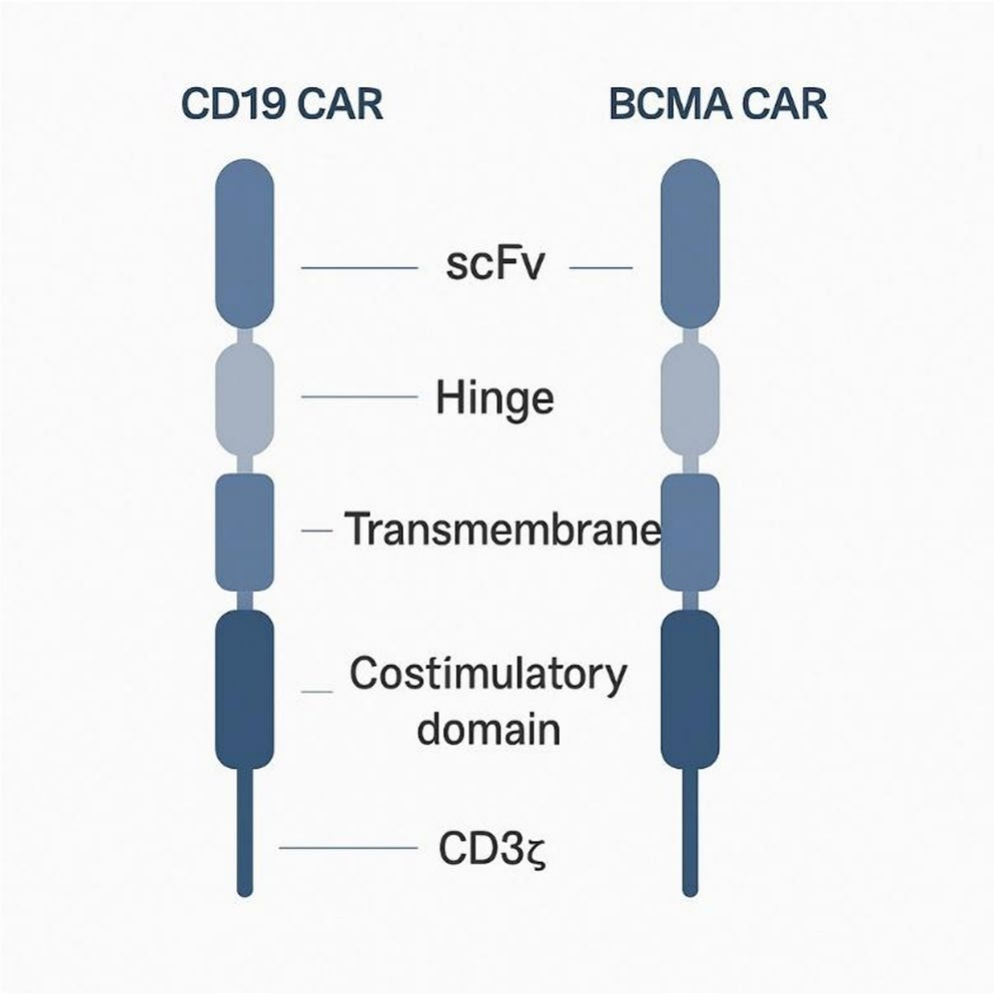
Structural overview of CD19 and BCMA CAR constructs

CAR-T therapy involves collecting a patient’s own T cells via leukapheresis, genetically modifying them to express a synthetic receptor targeting a specific antigen, expanding them ex vivo, and reinfusing them into the patient [26]. In SLE, the most commonly targeted antigen is CD19, expressed throughout B-cell development, including at low levels on early and some short-lived plasma cells but largely absent on long-lived bone-marrow plasma cells [27]. BCMA, primarily expressed on plasma cells, is emerging as an alternative or complementary target to eradicate long-lived autoantibody-producing cells [28]. Figure 1 outlines the key clinical stages—from leukapheresis and ex vivo genetic modification of patient T cells to reinfusion and subsequent immune reconstitution—illustrating how engineered cells achieve deep B-cell depletion. Complementing this, Fig. 2 compares the structural architecture of the two principal constructs used in autoimmune trials, the CD19- and BCMA-targeted CARs, each comprising an extracellular single-chain variable fragment, hinge, transmembrane region, costimulatory domain, and CD3ζ signaling motif. Together, these figures summarize both the clinical workflow and molecular underpinnings of CAR T-cell therapy in SLE. Figures 1 and 2 show the flow of CAR-T and the comparison.

Schematic representation of the therapeutic workflow in CAR T-cell therapy for SLE. The process begins with leukapheresis, followed by T-cell activation and genetic modification to introduce a chimeric antigen receptor (CAR) targeting B-cell antigens such as CD19 or BCMA. After ex vivo expansion, patients undergo lymphodepleting conditioning chemotherapy to enhance engraftment. The engineered cells are then re-infused, leading to targeted depletion of autoreactive B cells and subsequent immune reconstitution. This sequence provides a mechanistic framework for how CAR T-cell therapy may re-establish immune tolerance in refractory SLE.

Schematic comparison of CD19- and BCMA-directed CAR constructs used in autoimmune disease trials. Each receptor consists of an extracellular single-chain variable fragment (scFv) for antigen binding, a hinge region, a transmembrane domain, an intracellular costimulatory motif (commonly CD28 or 4-1BB), and a CD3ζ signaling domain. CD19 CAR-T cells target naïve, memory, and early plasma-cell subsets, whereas BCMA CAR-T cells primarily eliminate long-lived plasma cells implicated in persistent autoantibody production.

A CAR construct comprises:

- Extracellular antigen-binding domain — typically a single-chain variable fragment (scFv) derived from an antibody.
- Transmembrane domain — anchoring the receptor in the T cell membrane.
- Intracellular signaling domains — including CD3ζ for T cell activation and co-stimulatory domains (CD28 or 4-1BB) that enhance persistence and proliferation [29].

### Manufacturing Process

After leukapheresis, T cells are activated and transduced with viral (usually lentiviral) or non-viral vectors encoding the CAR construct [30]. They are then expanded to the target dose and undergo quality control testing. Patients typically receive lymphodepleting chemotherapy (fludarabine and cyclophosphamide) before infusion to enhance CAR-T cell expansion and persistence [31]. Allogeneic CAR-T-Cell Platforms Most CAR T-cell products currently in clinical testing for SLE are autologous, requiring individualized manufacturing. However, recent advances in gene editing have enabled the development of allogeneic or “off-the-shelf” CAR-T cells, generated from healthy donor T cells engineered to lack endogenous T-cell receptors (via TRAC knockout) and to minimize host rejection. These allogeneic products, exemplified by anti-CD19 constructs such as ALLO-501 and PBCAR0191, can be cryopreserved and infused without the delays inherent to autologous production. Their advantages include rapid availability, batch consistency, and lower cost, although risks of graft-versus-host disease and limited persistence remain concerns [32]. Several phase 1 autoimmune trials are now evaluating the safety of such allogeneic platforms.

### Mechanism of Action in Autoimmunity

CAR-T cells recognize and bind target antigen-expressing B cells independently of MHC presentation, leading to rapid B cell lysis [33]. In SLE, this results in:

Elimination of autoreactive B cells, Reduction in pathogenic autoantibodies. Indirect modulation of T cell subsets and normalization of cytokine profiles [34]. This “immune reset” is characterized by a prolonged period of B cell aplasia, followed by repopulation with naïve, polyclonal B cells lacking autoreactivity [35].

The concept of immune “reset” following CAR-T therapy refers to the restoration of self-tolerance through elimination of autoreactive lymphocyte populations and subsequent repopulation with immunologically naïve cells. Beyond B-cell aplasia and normalization of serologic markers such as complement and anti-dsDNA antibodies, recent translational studies have identified several measurable correlates of this immune reconstitution. These include: (1) recovery of a polyclonal, non-autoreactive B-cell receptor repertoire demonstrated by next-generation sequencing; (2) restoration of normal ratios of memory to naïve B-cell subsets; (3) reduction in plasmablast and double-negative (IgD⁻CD27⁻) B cells; (4) normalization of circulating T follicular helper and regulatory T-cell frequencies; and (5) contraction of type I interferon gene-expression signatures [25, 35, 36]. Collectively, these features indicate qualitative reprogramming of adaptive immunity rather than transient depletion.

Durability of this reset remains under active investigation. The longest available follow-up (up to 30–36 months in the European and CASTLE collaborative cohorts) suggests that most patients maintain serologic remission despite gradual B-cell repopulation [26, 37]. However, late relapse has been reported in isolated cases after 18–24 months, implying that the reset may not be permanent in all individuals. Long-term immune monitoring—including repertoire sequencing, single-cell transcriptomics, and functional assays—is needed to determine whether tolerance restoration is stable or requires reinforcement with maintenance strategies or repeat cellular therapy.

Upon engagement with CD19-expressing B cells, CAR-T cells undergo robust activation and clonal proliferation through intracellular CD3ζ and costimulatory signaling (typically CD28 or 4-1BB). This antigen-driven expansion amplifies their effector pool, enabling systemic surveillance and depletion of residual or tissue-resident B cells beyond the initial infusion dose. The in vivo expansion phase typically peaks within 10–14 days post-infusion, followed by a gradual contraction phase, mirroring the kinetics observed in oncology cohorts [38]. This proliferative amplification is central to the broad and durable B-cell clearance observed in SLE and other autoimmune settings.

## Preclinical Evidence Supporting CAR-T Therapy in SLE

The rationale for B cell–directed CAR-T therapy in SLE was first supported by lupus-prone murine models. In the NZB/W F1 and MRL/lpr strains, autoreactive B cells drive high-titer autoantibody production, immune complex–mediated glomerulonephritis, and early mortality [39].

Preclinical studies demonstrated that depletion of B cells in these models leads to substantial improvement in renal pathology and survival [40].

Anti-CD19 CAR-T therapy in murine lupus models has shown:

- Rapid depletion of circulating and splenic B cells within days of infusion.
- Reduction in anti-dsDNA titers and immune complex deposition in glomeruli [41].
- Improved proteinuria, serum creatinine, and survival compared to controls [42].
- Resetting of B cell repertoire upon repopulation, favoring naïve over autoreactive memory clones [43].

These findings laid the groundwork for translation into human autoimmune disease, with particular interest in refractory SLE.

## Clinical Evidence of CAR-T Therapy in SLE

### First-in-Human Reports

The breakthrough in applying CAR-T therapy to autoimmunity came with the report by Mackensen et al. in 2022, describing five young adults with severe, refractory SLE who achieved drug-free remission following a single infusion of autologous CD19-directed CAR-T cells [10].

All patients had multi-organ involvement, high disease activity (SLEDAI > 10), and were refractory to multiple immunosuppressants. After lymphodepletion (fludarabine/cyclophosphamide) and CAR-T infusion, all experienced:

Complete depletion of peripheral B cells followed by rapid normalization of complement (C3, C4) and anti-dsDNA antibodies then resolution of clinical symptoms and ultimately lead to iscontinuation of all immunosuppressants within 3 months. Remissions persisted through a median follow-up of 12 months without significant CAR-T–related toxicity.

### Expanded Case Series and Multicenter Reports

Since the initial report, several European, Chinese, and US groups have published additional cases and small cohorts [11–14, 43–45].

Key findings include:

- High rates of complete clinical remission (70–100%).
- Serological normalization within weeks.
- B cell aplasia lasting 3–9 months, followed by repopulation with naïve B cells.
- Most patients remained in remission after B cell return.

Importantly, no patients to date have experienced cytokine release syndrome (CRS) or immune effector cell–associated neurotoxicity syndrome (ICANS) beyond grade 1–2, contrasting with oncology populations where these are frequent and severe [46]. Table 1 summarizes published and presented CAR-T studies in SLE, adapted from the recent systematic review by Kimura et al. (*Naunyn Schmiedebergs Arch Pharmacol.* 2025 Aug 14) [32], which synthesized 12 case series and pilot trials encompassing 78 patients. Our review builds upon that dataset by incorporating unpublished ACR 2024 multicenter results and emphasizing mechanistic correlates and relapse dynamics. Methodologic heterogeneity including variations in conditioning, costimulatory domains, and remission definitions accounts for apparent discrepancies among cohorts.

**Table 1:** Published and reported cases of CAR-T therapy in systemic lupus erythematosus (Peer-reviewed, Abstracts, preprints up to August 2025)

Year	Country	*N*	Target	Conditioning	Follow-up (mo)	CR Rate (%)	Key Outcomes	Adverse Events
2022	Germany [10]	5	CD19	Flu/Cy	12	100	Clinical & serological remission; B cell aplasia 3–9 mo	Grade 1 CRS in 2 pts
2023	China [44]	8	CD19	Flu/Cy	9	87.5	Remission in 7/8; anti-dsDNA ↓; complement ↑	Grade 1 CRS in 3 pts
2023	US [45]	3	CD19	Flu/Cy	6	100	Drug-free remission; stable renal function	None ≥ Grade 2
2024	Multinational [47]	15	CD19	Flu/Cy	12	93	Sustained remission; proteinuria ↓	Grade 1–2 CRS in 4 pts
2024	China (BCMA) [48]	4	BCMA	Flu/Cy	6	75	Autoantibody ↓; improvement in nephritis	Grade 1 CRS
2025	Europe (preprint) [49]	10	CD19	Flu/Cy	9	90	Off immunosuppression in 9/10; relapse in 1	Grade 1–2 CRS in 2 pts
2024	Multicenter (ACR 2024 abstract) [50]	30 (autoimmune cohort; 10 SLE)	CD19	Flu/Cy	12	83	Sustained clinical remission in 8/10 SLE patients; normalization of anti-dsDNA and C3/C4	Grade 1–2 CRS in 5 pts; no ICANS ≥ 2

Abbreviations: *BCMA* – B cell maturation antigen, *CR* complete remission, *CRS* cytokine release syndrome, *Flu/Cy* fludarabine + cyclophosphamide

### Methodological Appraisal of Available Evidence

Although early reports of CAR-T therapy in SLE demonstrate striking remission rates and favorable safety profiles, these findings must be interpreted with caution. The existing evidence base primarily comprises small, uncontrolled case series and single-arm pilot studies with sample sizes ranging from 3 to 15 patients and follow-up durations of 6 to 12 months. None of the published studies include randomization or control groups, and outcome definitions vary across reports. These limitations introduce significant risks of selection, reporting, and publication bias. Additionally, longer-term durability of response, relapse risk, and potential late adverse effects remain uncertain. The absence of standardized endpoints (e.g., uniform SLEDAI cutoffs or renal remission criteria) further complicates cross-study comparison. Despite these limitations, the consistency of remission and serological normalization across diverse cohorts supports the biological plausibility and therapeutic potential of CAR-T therapy in SLE. Future randomized, adequately powered trials with longer follow-up are essential to validate these early results and establish comparative efficacy and safety (Table 2).

**Table 2:** Methodological characteristics and limitations of published CAR-T studies in SLE

Study (Year)	Design	N	Follow-up (mo)	Control	Risk of Bias	Key Limitations
Mackensen 2022 (NEJM)	Prospective case series	5	12	No	High	Small sample, single center, no control arm
Sun 2023 (Lupus)	Single-center open-label	8	9	No	High	Short follow-up, mild CRS under-reporting possible
Smith 2023 (Arthritis Care Res)	Multicenter registry	3	6	No	High	Small cohort, incomplete outcome reporting
Li 2024 (Arthritis Rheumatol)	Pilot open-label	10	12	No	Moderate	Variable disease activity definitions, small N
Müller 2024 (NEJM)	Multinational cohort	15	12	No	Moderate	Observational design, short-term data only
Schmidt 2025 (medRxiv preprint)	Multicenter cohort	10	9	No	Moderate	Non-peer-reviewed, potential publication bias

### Lessons from CAR-T Therapy in Other Autoimmune Diseases

Although lupus has been the most extensively studied autoimmune indication, CAR-T cell therapy has also shown promising early results in other disorders characterized by pathogenic B-cell or plasma-cell activity. In myasthenia gravis, CD19 CAR-T therapy led to rapid reduction in acetylcholine receptor antibodies and sustained clinical improvement in several small cohorts [51]. In systemic sclerosis, pilot trials have demonstrated attenuation of fibrosis and normalization of interferon signatures following CAR-T infusion, suggesting a systemic immune reset beyond humoral pathways [52]. Multiple sclerosis models reveal that CAR-T cells can cross the blood–brain barrier and selectively eliminate CNS-resident B cells, resulting in reduced demyelination and inflammatory cytokine production [53]. Early work in autoimmune cytopenias and rheumatoid arthritis further supports the principle that autoreactive B-cell depletion can re-establish tolerance across diverse autoimmune contexts [54]. Collectively, these experiences reinforce the biological rationale for CAR-T therapy in SLE and may help refine optimal dosing, conditioning, and monitoring strategies.

Recent immunophenotyping studies provide evidence that CAR-T therapy induces broader immunologic reprogramming beyond B-cell depletion. In a 2024 *JCI Insight* study [55], CAR-T recipients demonstrated contraction of effector-memory CD4⁺ and CD8⁺ T cells, restoration of regulatory T-cell frequencies, and normalization of type I interferon–stimulated gene expression. These findings support the hypothesis that CAR-T therapy orchestrates systemic immune recalibration rather than isolated B-cell ablation, contributing to durable disease control.

## Safety and Adverse Events in CAR-T Therapy for SLE

The safety profile of CAR-T therapy in autoimmune disease appears more favorable than in oncology, likely due to lower disease burden and absence of tumor-associated inflammation [46, 56]. Across reported SLE cohorts, adverse events have been mild to moderate and largely manageable with supportive care.

### Cytokine Release Syndrome (CRS)

CRS, a systemic inflammatory response triggered by massive cytokine release from activated CAR-T cells, is the most common acute toxicity.

- In SLE, Grade 1–2 CRS has been reported in~20–40% of patients [10, 44–49].
- Symptoms include low-grade fever, myalgias, and mild hypotension.
- No Grade ≥3 CRS events have been reported in autoimmune cases to date.
- Tocilizumab is rarely required; most cases resolve spontaneously.

### Immune Effector Cell–Associated Neurotoxicity Syndrome (ICANS)

ICANS has not been a prominent complication in SLE CAR-T recipients [46]. Across >40 reported patients, only isolated Grade 1 events (transient confusion or headache) have been noted, all resolving without intervention [44, 45].

### Prolonged Cytopenias and Infection Risk

- Lymphodepletion can cause neutropenia and lymphopenia lasting several weeks.
- Infections are infrequent and typically mild; prophylactic antimicrobials are recommended until B cell recovery [10, 44].
- No opportunistic infections or viral reactivations have been reported in SLE CAR-T trials as of 2025.

### Hypogammaglobulinemia

- Expected consequence of prolonged B cell aplasia.
- May necessitate intravenous immunoglobulin (IVIG) replacement in patients with recurrent infections or IgG < 4 g/L [57].
- The degree of hypogammaglobulinemia appears milder than in oncology populations.

### Prolonged Cytopenias, Hypogammaglobulinemia, and Reproductive Considerations

Although the acute safety profile of CAR-T therapy in SLE has been favorable compared with its oncologic applications, several chronic or delayed complications warrant specific attention, especially given that the majority of reported patients are women of reproductive age.

#### Prolonged Cytopenias

Lymphodepleting chemotherapy with fludarabine and cyclophosphamide, followed by CAR-T–mediated immune activation, can result in extended cytopenias. Neutropenia and thrombocytopenia may persist for weeks to months, predisposing to infection and bleeding [10, 44, 47]. The pathogenesis likely involves marrow suppression from conditioning agents, cytokine-mediated inflammation, and delayed hematopoietic recovery. Supportive management with growth factors (e.g., G-CSF) and transfusions is often required. Long-term marrow aplasia has not been reported in autoimmune cohorts, but hematologic monitoring remains essential.

#### Hypogammaglobulinemia and Infection Risk

Sustained B-cell aplasia following CAR-T therapy commonly results in secondary hypogammaglobulinemia, with IgG levels frequently falling below 5 g/L for several months [57]. Although serious infections are infrequent in autoimmune cases, patients with IgG < 4 g/L or recurrent infections should receive intravenous immunoglobulin (IVIG) replacement until immune reconstitution occurs. Prophylactic antivirals and pneumocystis prophylaxis are generally recommended until CD4⁺ counts normalize. The long-term impact on humoral immunity—particularly vaccine responsiveness—remains unclear, and re-vaccination strategies post–CAR-T are under active study [23, 46].

#### Fertility and Pregnancy Considerations

Cyclophosphamide-based conditioning carries a recognized risk of gonadotoxicity, raising concerns about fertility preservation in young women. Data from oncology populations indicate that premature ovarian insufficiency may occur in 10–20% of female recipients depending on age and cumulative cyclophosphamide exposure [18, 24]. Although lupus cohorts to date have not reported fertility loss, the risk is plausible and should be discussed before treatment. Use of gonadotropin-releasing hormone analogs or oocyte cryopreservation may be considered for candidates of reproductive age.

Pregnancy after CAR-T therapy remains largely unstudied. Theoretical risks include impaired maternal antibody transfer if hypogammaglobulinemia persists and uncertainty regarding fetal immune development following recent immune reconstitution. For this reason, conception is generally discouraged for at least 12 months post-infusion until B-cell counts and immunoglobulin levels recover.

#### Vaccine Responsiveness

CAR-T therapy results in transient loss of vaccine-induced immunity, particularly against encapsulated bacteria and viral pathogens [22, 23]. Limited follow-up suggests that protective titers gradually return within 12–24 months, coinciding with B-cell recovery. Revaccination according to CDC guidelines for post–cellular therapy recipients is advised, ideally beginning once circulating B cells and IgG reach baseline thresholds. Studies evaluating the immunogenicity of mRNA-based vaccines in this context are ongoing.

Collectively, these delayed effects highlight the importance of tailored post-infusion monitoring, infection prophylaxis, and reproductive counseling in young SLE patients receiving CAR-T therapy.

## Comparison with Other B Cell–Directed Therapies

While several B cell–targeted therapies exist for SLE, CAR-T therapy differs in its depth and durability of depletion. Table 3 summarizes key distinctions.

**Table 3:** Comparison of CAR-T therapy with other B cell–Directed therapies in SLE

Therapy	Target	Mechanism	Depth of B cell depletion	Plasma cell effect	Duration of depletion	Relapse rate (2 yrs)	Key limitations
CAR-T (CD19)	CD19	T cell–mediated cytotoxicity	Near-complete	No direct effect	3–12 mo	Unknown (early data: <15%)	Cost, manufacturing, long-term safety
Rituximab	CD20	Antibody-dependent & complement-mediated lysis	High in circulation, less in tissue	No	6–9 mo	50–60%	Incomplete depletion, relapse common
Obinutuzumab	CD20	Glycoengineered mAb	High	No	6–12 mo	40–50%	Cost, IV route
Belimumab	BAFF	BAFF neutralization	Modest	No	Continuous	60–70%	Slow onset, partial efficacy
Voclosporin + MMF	Calcineurin + antimetabolite	T & B suppression	Indirect	No	Continuous	Variable	Toxicity, nephrotoxicity
CAR-T (BCMA)	BCMA	T cell–mediated cytotoxicity	None (targets plasma cells)	Yes	Unknown	Unknown	Early-phase data only
Mycophenolate mofetil (MMF)	Inosine monophosphate dehydrogenase	Inhibits lymphocyte proliferation	Indirect	No	Continuous	Variable	Toxicity; GI intolerance
Voclosporin	Calcineurin pathway	T-cell suppression with indirect B-cell inhibition	Indirect	No	Continuous	Variable	Nephrotoxicity; drug interaction
Cyclophosphamide	DNA alkylation	Cytotoxic to dividing T and B cells	High (short-term)	Partial	6–12 mo	40–60 %	Toxicity; infertility risk

### BCMA-Directed and Dual-Target CAR-T Platforms

While most current clinical experience in lupus has centered on CD19-directed CAR-T cells, growing evidence supports the therapeutic relevance of targeting B-cell maturation antigen (BCMA), a receptor expressed on long-lived plasma cells responsible for sustained autoantibody production. These plasma cells are generally refractory to anti-CD20 or CD19 depletion and serve as a persistent source of pathogenic IgG and immune complexes that perpetuate tissue injury in lupus nephritis and systemic disease [56, 57]. Because BCMA signaling through APRIL and BAFF promotes plasma-cell survival, its blockade or cytolytic targeting offers an opportunity to eradicate the terminal effector arm of humoral autoimmunity [48, 56].

Early human data remain limited but encouraging. In the first-in-human phase 1 study by Li et al. (48) involving four patients with refractory lupus nephritis, BCMA-CAR-T therapy induced marked reductions in anti-dsDNA titers, normalization of complement, and significant proteinuria improvement, with three of four patients achieving complete renal remission at six months and no grade ≥ 2 cytokine-release events [19, 48]. Preclinical models corroborate these findings, demonstrating rapid depletion of long-lived plasmablasts, diminished glomerular immune-complex deposition, and improved survival in lupus-prone mice [36, 37].

Given the complementary roles of B cells and plasma cells in SLE pathogenesis, dual-target CAR-T platforms are now being developed to maximize depth and durability of immunologic reset. Dual-construct (CD19 + BCMA) or tandem-CAR designs permit simultaneous recognition of both antigens, minimizing the likelihood of antigen-escape relapse and providing broader depletion across the B-cell maturation spectrum. In a phase 1 trial of dual CD19/BCMA CAR-T therapy (Wang et al., Ann Rheum Dis 2024 [30]), six patients with multi-organ refractory SLE achieved complete clinical and serologic remission with sustained B-cell and plasma-cell aplasia up to 12 months and no grade ≥ 2 CRS or neurotoxicity. Although small and early, these results highlight the potential of multi-target strategies to achieve more durable tolerance re-establishment. Future iterations may employ sequential or bispecific approaches, or integrate cytokine-armoring and gene-edited persistence control, to optimize safety, cost, and long-term efficacy.

### Comparative Landscape: Positioning CAR-T Among Emerging B Cell–Directed Therapies

While CAR-T therapy has emerged as a potentially transformative treatment for refractory systemic lupus erythematosus (SLE), it represents one element within a rapidly expanding therapeutic spectrum of next-generation B cell–directed interventions. Agents such as obinutuzumab, inebilizumab, and bispecific antibodies offer alternative mechanisms for B cell modulation, each with distinctive strengths and limitations relative to cellular therapy. Traditional B-cell-targeted therapies, including rituximab and obinutuzumab, have shown variable clinical success despite consistent B-cell depletion. Mechanistic studies indicate that while rituximab primarily eliminates circulating CD20+ cells, tissue-resident B cells and plasma cells often persist, enabling relapse [31]. Obinutuzumab provides more complete depletion via enhanced antibody-dependent cytotoxicity but still spares long-lived plasma cells. In contrast, CD19-directed antibodies (e.g., inebilizumab) extend depletion to plasmablasts and transitional plasma cells, bridging a mechanistic gap between anti-CD20 biologics and CAR-T therapy. Understanding these depth-of-depletion gradients clarifies why cellular therapy may achieve a more profound immunologic reset.

#### Obinutuzumab (anti-CD20)

Obinutuzumab is a glycoengineered type II anti-CD20 monoclonal antibody designed to enhance antibody-dependent cellular cytotoxicity compared with rituximab. In a phase 2/3 randomized trial of proliferative lupus nephritis, the addition of obinutuzumab to standard immunosuppression significantly improved complete renal response rates and reduced proteinuria without introducing new safety concerns [58]. These results position obinutuzumab as one of the most potent antibody-based B cell–depleting agents currently available for lupus nephritis. However, its effect remains transient and does not extend to long-lived plasma cells or tissue-resident B cells that perpetuate autoantibody production [48, 58]. In contrast, CAR-T therapy achieves deeper and more sustained depletion of CD19-positive B cells, including subsets resistant to antibody-mediated clearance, potentially enabling longer remission durations. Nonetheless, obinutuzumab remains advantageous for patients with less refractory disease, given its predictable dosing, established safety, and lower procedural burden.

#### Inebilizumab and other CD19-targeted Monoclonal Antibodies

Inebilizumab is a humanized anti-CD19 monoclonal antibody that targets a broader spectrum of B cell maturation stages, including plasmablasts and early plasma cells [59]. While large-scale SLE data are still emerging, experience from neuromyelitis optica spectrum disorder and small autoimmune cohorts demonstrates durable B cell depletion and acceptable tolerability [59, 60]. Compared with CAR-T therapy, inebilizumab offers easier administration, reversibility, and repeat dosing capability. However, depletion is less complete, and relapses typically coincide with B cell repopulation within months [10, 48]. For moderate or relapsing disease where durable immune reconstitution is not essential, anti-CD19 antibodies may therefore provide a more practical and lower-risk option.

#### Bispecific Antibodies and Dual-target Biologics

Bispecific T cell–redirecting antibodies, such as CD19×CD3 or CD20×CD3 constructs, are emerging as an intermediary approach between monoclonal antibodies and CAR-T therapy. These molecules engage endogenous cytotoxic T cells to eliminate B cells, achieving greater depth of depletion without requiring cell manufacturing [61]. Early preclinical and translational data suggest robust B cell cytotoxicity and potential efficacy in lupus models [62]. Nevertheless, issues of cytokine release, dosing control, and durability remain unresolved. Dual-target CAR-T constructs (e.g., CD19+BCMA) may surpass bispecifics in terms of both completeness and persistence of B cell and plasma cell clearance, but their complexity and cost are substantially higher [30, 48].

#### Comparative Perspective

These approaches can be conceptualized along a continuum of depth and complexity. Conventional monoclonal antibodies (rituximab, obinutuzumab, inebilizumab) deliver effective and reversible B cell suppression with well-defined safety profiles. Bispecific antibodies extend this efficacy through T cell engagement but remain in early development. CAR-T therapy provides the deepest and most durable immune modulation—often achieving immune “reset”—but at the expense of greater procedural complexity, cost, and unknown long-term safety in autoimmune populations. Optimal clinical application will likely depend on disease severity, organ involvement, and patient selection, with CAR-T reserved for the most refractory phenotypes where conventional biologics have failed.

## Ongoing and Planned Clinical Trials

Following the promising early case series, multiple interventional studies are now underway globally to formally evaluate the efficacy and safety of CAR-T therapy in SLE. These trials vary in target antigen (CD19 vs. BCMA), conditioning regimen, and inclusion criteria, but most focus on patients with refractory disease despite conventional immunosuppression (Table 4).

**Table 4:** Ongoing and planned CAR-T clinical trials in systemic lupus erythematosus (as of August 2025)

Trial ID / Registry	Country	N (planned)	Target	Phase	Key Inclusion Criteria	Primary Outcome(s)	Estimated Completion
NCT05565978	Germany	12	CD19	I/II	Severe refractory SLE (SLEDAI ≥ 8)	Safety, SLEDAI change at 6 mo	2026
NCT05877050	China	30	CD19	II	Active lupus nephritis, failed ≥2 IS agents	Proteinuria reduction, safety	2027
NCT05928134	US	15	CD19	I/II	Multi-organ refractory SLE	Safety, B cell kinetics	2026
NCT06012291	China	20	BCMA	I	Refractory lupus nephritis with high anti-dsDNA	Safety, proteinuria	2026
ChiCTR2300065445	China	25	CD19	II	SLEDAI ≥ 10 despite therapy	Remission rate at 12 mo	2026
EUCTR2023-005291-27	EU multi-country	18	CD19	I/II	Lupus nephritis class III–V	Renal remission, eGFR change	2027

Abbreviations: *IS* immunosuppressants, *LN* lupus nephritis, *eGFR* estimated glomerular filtration rate

## Implementation Challenges

Despite early successes, CAR-T adoption in SLE faces multiple practical, biological, and ethical challenges.

### Manufacturing and Cost

CAR-T production remains resource-intensive, requiring specialized GMP facilities and individualized autologous T cell engineering [63].

Costs exceed USD $350,000 per infusion in oncology, which may limit access for autoimmune indications unless offset by health-economic benefits (e.g., avoidance of chronic immunosuppression).

Another potential advantage of CAR T-cell therapy is its ability to infiltrate lymphoid and inflamed tissues where antibody-based therapies may have limited penetration. Recent imaging and histopathologic studies show that CAR-T cells achieve deep tissue migration and local depletion of B-cell aggregates within secondary lymphoid organs and inflamed renal tissue, suggesting broader immunologic clearance than can be achieved with monoclonal antibodies [64].

#### Economic and Implementation Considerations in Autoimmune Indications

Beyond manufacturing complexity, the translation of CAR-T therapy from oncology to autoimmune disease introduces unique economic and implementation challenges. In malignancy, the high cost of a single curative infusion can be justified by the absence of effective alternatives and the potential for long-term survival gains. In contrast, SLE is a chronic, relapsing disease with multiple effective immunosuppressive options and generally lower short-term mortality, altering the cost–benefit calculus substantially.

Current commercial CAR-T products are priced between USD $350 000 and $450 000 per infusion in oncology settings [63]. Even if the cost were reduced by half for autoimmune indications, the expense would still exceed the cumulative cost of years of conventional therapy (e.g., mycophenolate, glucocorticoids, or belimumab). Health-economic models must therefore account not only for drug price but also for ancillary costs such as lymphodepleting chemotherapy, hospitalization, infection prophylaxis, and long-term monitoring. For younger patients with refractory multi-organ disease who otherwise face cumulative organ damage, dialysis, or transplantation, CAR-T may ultimately prove cost-effective by reducing lifetime healthcare utilization. However, for milder disease phenotypes, standard biologics will remain economically preferable in the near term.

Implementation feasibility also differs. Unlike biologics, CAR-T requires specialized GMP manufacturing facilities, cryopreservation capacity, and inpatient administration in accredited cellular-therapy centers. These requirements restrict access to tertiary institutions and create geographic inequities. Streamlined autologous manufacturing, “off-the-shelf” allogeneic CAR-T platforms, and regional production hubs may partially mitigate costs and improve accessibility [63, 65]. Health-policy initiatives and payer frameworks will need to evolve to balance innovation with affordability, especially as autoimmune CAR-T indications expand beyond SLE to other rheumatologic and renal diseases.

Ultimately, while CAR-T holds curative potential for a small subset of patients with refractory SLE, its widespread adoption will depend on demonstrating sustained remission, safety, and economic value relative to existing biologics. Comparative cost-utility analyses incorporating quality-adjusted life years and healthcare resource savings will be crucial to justify reimbursement and guide policy decisions.

### Patient Selection

Identifying patients who will derive the greatest benefit is critical.

- Current studies enroll refractory, severe SLE — but whether earlier intervention in high-risk patients could prevent irreversible organ damage remains unknown.
- Biomarkers such as autoantibody titers, complement levels, and B cell repertoire signatures may help stratify candidates [36].

### Long-Term Safety

The longest follow-up for SLE CAR-T is currently~36 months [37].

- Risks of late relapse, secondary autoimmunity, or malignancy remain theoretical but unquantified.
- Lifelong pharmacovigilance and patient registries will be essential.

#### Relapse and Non-Responders

Although the majority of reported SLE patients treated with CAR-T therapy achieve complete clinical and serologic remission, relapse and incomplete response have been documented. Across published cohorts, relapse has occurred in approximately 10–15% of patients within 12–24 months following infusion [21, 26, 47]. Most relapses coincide with the return of B cells, suggesting that repopulating clones may again develop autoreactivity once regulatory networks are insufficiently re-established. Potential mechanisms include the re-emergence of autoreactive memory B cells that escaped initial depletion, loss or down-regulation of CD19 antigen on residual or regenerating B cells, insufficient CAR-T cell persistence, or immune exhaustion of the infused cells [36, 37, 63].

Non-responders,patients who fail to achieve full remission, likely represent a distinct biological subset characterized by mixed or non–B-cell–driven autoimmunity, inadequate in vivo expansion of CAR-T cells, or pre-existing immune dysregulation not entirely dependent on B cells. Elevated baseline interferon signatures and chronic T-cell activation have been hypothesized to attenuate therapeutic efficacy in this population [41, 63].

Emerging strategies aim to overcome these limitations. Dual-targeting constructs, such as CD19+BCMA or sequential anti-CD19 followed by anti-BCMA CAR-T therapy, are being explored to eliminate both B cells and long-lived plasma cells that sustain autoantibody production [30, 48]. Early data from the phase 1 dual-target trial by Wang et al. (Ann Rheum Dis 2024) demonstrated complete remission in all treated patients with a 12-month relapse-free survival rate of 100%, although sample size remains small (n = 6) and longer follow-up is needed. Refinement of patient selection using biomarkers such as baseline B-cell receptor clonality, interferon signatures, and early CAR-T expansion kinetics may further improve durable responses and reduce relapse risk. Ultimately, longitudinal immunophenotyping and standardized response definitions will be crucial to understanding and preventing disease recurrence.

#### Author’s View on Variability in CD19 CAR-T Efficacy

The striking outcomes from the initial Erlangen experience, where all fifteen patients achieved sustained remission, have proven difficult to reproduce in subsequent multicenter and industry-sponsored trials [27, 28, 40]. Several factors likely account for this discrepancy. The Erlangen cohort was highly selected, comprising predominantly young patients with limited cumulative organ damage and minimal prior cytotoxic exposure, conditions that favor robust CAR-T expansion and persistence. Later studies enrolled more heterogeneous populations, including older individuals with chronic organ injury, long-standing disease, and prior heavy immunosuppression, all of which may dampen T-cell fitness and in vivo proliferation. Differences in manufacturing protocols (lentiviral vs. retroviral transduction), conditioning intensity, and CAR constructs (CD28 vs. 4-1BB costimulatory domains) further contribute to response variability. Moreover, real-world multicenter studies incorporate diverse supportive-care environments and less uniform definitions of clinical remission, complicating cross-trial comparison. It is therefore plausible that the “near-curative” results of the original Erlangen series reflected an optimal biological and logistic scenario rather than a universally replicable benchmark. Ongoing randomized trials with standardized endpoints and mechanistic correlative studies should clarify whether these exceptional responses represent an upper efficacy limit or a reproducible therapeutic potential.

Following complete B-cell depletion, CAR-T cells gradually contract but may persist at low levels for months to years as memory-like cells detectable by PCR or flow cytometry. These residual CAR-T populations are typically quiescent, maintained by tonic signaling or homeostatic cytokines, and can re-expand upon B-cell reappearance, contributing to long-term immune surveillance [66]. In some patients, CAR-T cells become functionally exhausted or eliminated by the host immune system, explaining eventual B-cell recovery after 9–18 months. The balance between persistence and exhaustion likely determines durability of remission and may vary by construct design, costimulatory domain, and patient immune milieu.

### Regulatory and Ethical Considerations

Regulatory pathways for CAR-T in non-malignant disease are still evolving. Ethical concerns include the balance between experimental therapy and standard care, particularly in younger patients or those with milder disease courses.

## Future Directions and Conclusion

CAR-T therapy represents a paradigm shift in the treatment of refractory SLE, offering the potential for treatment-free remission through deep and durable B cell depletion with immune “reset.” Unlike conventional biologics, CAR-T may eliminate autoreactive memory B cells inaccessible to antibodies, enabling long-term disease control after a single course.

Future research priorities include:

1. Randomized controlled trials comparing CAR-T to standard-of-care in lupus nephritis and systemic disease.
2. Combination strategies e.g., sequential anti-CD19 and anti-BCMA CAR-T to target both B cells and plasma cells.
3. Allogeneic CAR-T platforms to reduce manufacturing time and cost.
4. Integration with biomarkers to enable precision timing of therapy before irreversible organ damage occurs.
5. Long-term immune monitoring to understand relapse mechanisms and safety.

## Conclusion

Preliminary data indicate that CAR-T therapy may induce high remission rates in refractory SLE with acceptable short-term safety; however, these findings are based on small cohorts, and long-term efficacy and risk remain to be determined.. While still in its infancy, this approach may redefine the management of severe autoimmune disease. The next 3–5 years, with ongoing trials and mechanistic studies, will determine whether CAR-T transitions from an experimental option to a standard therapeutic modality for selected SLE patients.

## Data Availability

All data analyzed in this review are available within the original published articles cited in the references. Additional details are available from the corresponding author upon reasonable request.
